# Supplementary material for: Megakaryocytes possess a STING pathway that is transferred to platelets to potentiate activation
Source: Life Sci Alliance. 2023 Nov 22;7(2):e202302211. doi: 10.26508/lsa.202302211 (PMC10665521; doi:10.26508/lsa.202302211)
Supplement: Supplementary file 1 [file LSA-2023-02211_TableS1.docx]

Table S1: List of primers

| mccl5 FWD | GCTGCTTTGCCTACCTCTCC |
| --- | --- |
| mccl5 rev | TCGAGTGACAAACACGACTGC |
| mcGAS-RT-FWD | TGAACATGTGAAGATTTCTGCTCC |
| mcGAS-RT-REV | TGACTCAGCGGATTTCCTCG |
| mISG15_FWD | CAATGGCCTGGGACCTAAAG |
| mISG15_REV | TAAGACCGTCCTGGAGCACT |
| mIfit1-p56-RT-FWD | GAGAGTCAAGGCAGGTTTCT |
| mIfit1-p56-RT-REV | TCTCACTTCCAAATCAGGTATGT |
| ifitm3 FWD Set 1 | GGTCTGGTCCCTGTTCAATAC |
| ifitm3 REV Set 1 | GTCACATCACCCACCATCTT |
| mIFNB For | CCCTATGGAGATGACGGAGA |
| mIFNB rev | CCCAGTGCTGGAGAAATTGT |
| RSAD2-FWD | CTGTGCGCTGGAAGGTTT |
| RSAD2-REV | ATTCAGGCACCAAACAGGAC |
| TBP-mouse_F1 | AATAAGAGAGCCACGGACAAC |
| TBP-mouse_R1 | TCTGGATTGTTCTTCACTCTTGG |
